# Survivor perspectives on research priorities for assessing mental health outcomes after school shootings: a qualitative study

**DOI:** 10.1186/s40621-025-00570-4

**Published:** 2025-03-12

**Authors:** Camerin A. Rencken, Kelsey Conrick, Isaac C. Rhew, Carol A. Davis, Ali Rowhani-Rahbar

**Affiliations:** 1 Department of Epidemiology, University of Washington, 3980 15th Ave NE, Seattle, WA 98195 USA; 2 Firearm Injury & Policy Research Program, University of Washington, Seattle, WA USA; 3 Department of Psychiatry and Behavioral Sciences, University of Washington, 2815 Eastlake Ave E Suite 200, Seattle, WA 98102 USA; 4 College of Education, University of Washington, Miller Hall, 2012 Skagit Ln, Seattle, WA 98105 USA; 5 School Mental Health Assessment, Research, and Training Center, University of Washington, Seattle, WA USA

**Keywords:** Firearms, School shootings, Mental health, Participant-led research agenda

## Abstract

**Background:**

Firearm violence is a major public health problem and the leading cause of death among children and youth aged one to nineteen in the United States (US). School shootings, though a relatively rare form of firearm violence in the US, have been occurring with increasing frequency, exposing more than 380,000 students to such events since 1999. This study engaged school shooting survivors to identify key research areas regarding their mental health, aiming to enhance the relevance and impact of future research for this community.

**Methods:**

Participants for individual and group interviews were recruited from survivor support groups and through snowball sampling between May and August 2024. The interview guide, based on a recent scoping review highlighting gaps in research on the mental health impacts of school shootings, facilitated discussions on participants’ experiences, needs, and research priorities. Interviews were recorded, transcribed verbatim, and analyzed using thematic analysis. Thirteen individuals participated (median age: 40 years; range: 18–47), including 11 former student survivors, one parent of a survivor, and one sibling of a victim. These participants represented ten school shootings from 1997 to 2022 across eight US states including Colorado, Florida, Kentucky, Maryland, Michigan, Oregon, Tennessee, and Washington. Eight participants experienced a mass school shooting (four or more fatalities excluding the perpetrator).

**Results:**

The study identified three key research priorities: (1) understanding the long-term mental health impacts of school shootings across the life course, (2) expanding research to include broader outcomes beyond traditional mental health metrics, and (3) diversifying research approaches, study designs, and study populations to better capture the varied experiences of survivors.

**Conclusion:**

There is a need for researchers to explore a wider range of outcomes, communities, and timeframes when studying the mental health impacts of school shootings. Such investigations are essential for understanding the complex and unique aspects of recovery and resilience among survivors. Centering survivor perspectives enhances our understanding of ongoing challenges facing survivors of school shootings, which should be prioritized in designing and evaluating interventions and policies.

## Introduction

Firearm violence is a major public health problem and the leading cause of death among children and youth aged one to nineteen in the United States [1]. School shootings, while rare compared to other forms of firearm violence, are a critical part of this epidemic, and their frequency has been increasing over the last two decades [2]. In 2023, there were 160 incidents of firearms being discharged on school grounds, resulting in 46 deaths and 106 injuries— with each tragedy impacting entire communities [3]. Moreover, estimates suggest that since the Columbine High School tragedy in 1999, more than 383,000 students have experienced firearm violence at school.

While the number of school shootings appears to have increased over the last two decades [2], the long-term mental health impacts of school shootings are not well understood [4]. A scoping review on youth firearm violence exposure found 14 studies addressing mass or school shootings, with only five focusing on mental health outcomes [5]. Among the limited research on outcomes beyond the immediate aftermath, findings have linked various forms of exposure to school shootings to emotional distress, increased stress-related emergency visits among youth, and higher antidepressant use among youth at affected schools [6–8]. Additionally, research on Sandy Hook Elementary School and Columbine High School reported increased absenteeism and lower test scores among affected students [9]. A national-level study also associated exposure to school shootings with declines in overall health and well-being [10]. These studies were able to examine long-term outcomes by utilizing data sources such as medical records and academic performance, enabling research that, in some cases, tracked impacts over several years. However, such studies remain scarce. Further, although existing studies provide valuable insights into the mental health impacts of school shootings, there is potential for future work in the field to center the voices of affected communities in designing research questions that align with the needs of the community [11]. 

Research that does not incorporate the perspectives of survivors risks marginalizing their experiences and overlooking their needs, which is particularly of concern in this context given that school shootings disproportionately affect students and communities of color [11–13]. This exclusion can hinder the identification of contextually-specific factors influencing mental health after school shootings, ultimately limiting the development of effective interventions and policies tailored to support communities [11]. Prior medical studies have indicated that the priorities of patients often differ from the priorities identified by experts and/or their physicians, highlighting the importance of ensuring research questions originate from patients and survivors themselves [11, 14–16]. One method increasingly used to address this issue is research-priority setting, which engages stakeholders in identifying key research areas [11, 17]. This approach involves collaboratively identifying research priorities with the greatest potential for public benefit [18]. It has been particularly effective in low-resource settings where careful prioritization of resource allocation is essential [19]. However, to our knowledge, this approach has not been applied within the community of survivors of school shootings. Therefore, this study involved survivors in discussions to explore gaps in the literature and provide opportunities for them to suggest new areas of inquiry. The goal was to develop participant-driven recommendations for future research priorities, grounded in the lived experiences of school shooting survivors.

## Methods

###  Study design and sample selection

This study employed a qualitative, phenomenological approach to understand and describe the lived experiences of individuals who survived school shootings [20]. Study selection criteria included being age 18 or older, ability to provide informed consent, and self-identifying as a survivor of a school shooting. This included individuals who were either physically present at the school during the event or had an immediate family member (e.g., parent or sibling) at the school during the event. Screening was conducted prior to the interview to ensure that participants were survivors of school shootings who met such criteria. A combination of individual and group interviews was conducted to capture a wide range of perspectives (*n* = 6 and *n* = 2; respectively). The study population consisted of 13 participants (median age: 40 years; range: 18–47), with the majority identifying as female (92%) and White (85%), and 23% identified as Asian or Asian American. Most participants (54%) resided in suburban areas at the time of the interview, with smaller proportions from urban (38%) and rural (8%) neighborhoods. (Table 1) In terms of the school shootings, 8 of the 13 participants experienced a mass school shooting (involving four or more deaths not including the perpetrator, as defined by a few sources including the Gun Violence Archive [21]). The incidents occurred between 1998 and 2022 in eight states including Colorado, Florida, Kentucky, Maryland, Michigan, Oregon, Tennessee, and Washington. (Table 1) Nine of the shootings occurred at high schools, while one took place at a community college.

**Table 1:** Participant demographics (*n* = 13)

	Frequency (%) *
**Sex**	
Male	1 (8%)
Female	12 (92%)
**Median age**, years	40 years (Range: 18–47)
Self-identified race*	
Asian or Asian American	3 (23%)
Black or African American	1 (8%)
White	11 (85%)
**Participants’ current residential neighborhood**	
Urban	5 (38%)
Suburban	7 (54%)
Rural	1 (8%)
Non-mass school shooting incident**	5 (38%)
**Year of shooting**	
1990–2005	6 (46%)
2006– Present	7 (54%)

*Numbers sum to more than 100% because of non-exclusive categories; **Mass shooting defined as four of more deaths including the perpetrator. [21]

### Data collection

Participants were recruited using snowball sampling, with outreach through two survivor support groups and community leaders. Recruitment was designed to protect privacy and confidentiality, utilizing an opt-in approach where potential participants were provided information about the study and invited to contact the research team voluntarily. Participants were reimbursed $75 USD for their time. Interviews were conducted via videoconferencing on Zoom between May and August 2024, lasting between 45 and 90 min each. CR led the interviews, supported by KC, using semi-structured guides developed from a comprehensive scoping review on school shootings and mental health conducted by our team (Appendix 1). These guides were iteratively reviewed and refined by subject matter and methodological experts including scholars and practitioners specializing in school emergency responses identified through professional networks and academic referrals. The interview guides allowed participants to share additional insights or suggest research priorities that they felt were important. The included questions were created by identifying the three most significant gaps in the literature, which were then presented to the participants. For each gap, we asked participants about the importance of addressing it and what additional research should include to further the field.

### Data analysis

The data were analyzed using an abductive analytic approach, which integrates both inductive and deductive methods [22, 23]. This approach allowed for the identification of new, unanticipated themes emerging from participant responses (inductive), while also ensuring that pre-determined categories from the existing literature were systematically examined (deductive). The final results included research agenda items that were supported by both the gaps identified in the literature review and the endorsement of these items by a majority of participants. All interviews were transcribed verbatim, and a codebook, closely aligned with the semi-structured interview guide, was iteratively developed and refined. This process involved continuously reviewing the data and adjusting the codebook to better capture emerging patterns and insights. Additional codes were added based on insights during interim analyses, and transcripts were hand coded. Following preliminary analyses, all participants were invited to a videoconference where initial findings were presented. During this session, participants had the opportunity to reflect on and refine the results. Input from this meeting was incorporated into the final results, further enhancing the reliability of the findings.

### Ethical review

This research was deemed exempt by the University of Washington Institutional Review Board as it involved no more than minimal risk to the subjects. Informed consent was obtained from all participants prior to their interviews, and permission was granted to audio record the sessions. The audio tapes were stored on a password-protected and encrypted drive to ensure security. Additionally, none of the transcripts contained names or identifying information, and care was taken to ensure that any mention of the school, city, or other identifying details were either omitted or anonymized during the transcription process.

## Results

The present qualitative study identified three key research priorities: (1) understanding the long-term mental health impacts of school shootings across the life course, (2) expanding research to include broader outcomes beyond traditional mental health metrics, and (3) diversifying research approaches, study designs, and study populations to better capture the varied experiences of survivors (Table 2).

**Table 2:** Participant-identified research priorities

Research priorities	Proposed research directions	
Research priority 1: Understand the mental health impacts across the life course	• Investigate persistent long-term mental health challenges faced by survivors over time • Explore mental health challenges that emerge after the acute phase of recovery • Examine the non-linear trajectories of mental health challenges and recovery processes among survivors	
Research priority 2: Include comprehensive outcomes beyond traditional mental health metrics	• Assess the impact of school shootings on social dynamics and interpersonal relationships • Evaluate the influence of trauma on survivors’ life goals, aspirations, and motivation • Investigate the relationships between school shooting exposure and substance use • Analyze the financial implications of trauma and recovery for survivors and their families • Examine the effects of school shootings on academic performance and educational outcomes	
Research priority 3: Diversify research approaches and study designs	Study populations	• Include diverse perspectives from parents, teachers, siblings, and first responders in research • Investigate the unique experiences and needs of boys and men in the context of school shootings
	Study design	• Methods: incorporate additional qualitative research methods to capture lived experiences • Type of school shooting: expand research to include a broader range of school shootings, including non-mass incidents

### Research priority 1: understand the mental health impacts across the life course

Research Priority 1, focusing on the long-term mental health impacts of school shootings, emerged as a critical issue for many participants. They consistently reported enduring psychological challenges that extended far beyond the immediate aftermath of the event. Recovery from school shootings is often non-linear, marked by periods of progress and setbacks. As one participant shared, “It’s definitely gotten better over time… but if [the sound] the right frequency and the right pitch… it’ll still have the same reaction that I did back then.” Despite improvements in managing trauma, certain triggers—such as specific sounds or reminders—continue to reactivate distress, causing reactions that feel as acute as in the immediate aftermath. As another participant reflected, “A siren somewhere nearby, and I just got so viscerally freaked out, like I was back in school.”

Furthermore, some trauma-related effects did not become apparent until years later, adding to the complexity of recovery and emphasizing the need for longitudinal research. One participant described the dynamic nature of mental health trajectories: “People change over the years with how they exhibit it… it’s fluid.” This reinforces the need for ongoing measurement of symptoms such as anxiety or PTSD over time. As one participant observed, “I think the first two months, I pretty much treated it like a school break… but I think as time has gone on, the anxiety I feel about the situation has definitely increased.” The delayed intensification of mental health challenges underscores the importance of assessing outcomes at multiple time points, as suggested by participants.

In addition to psychological impacts, many survivors reported persistent physical health issues that emerged long after the shooting. One participant noted, “The long-term health ramifications on the body… you can’t even feel it until year two, when things start to settle in,” highlighting that physical symptoms often surface after the initial trauma. Issues like fatigue, headaches, and other stress-related ailments became regular challenges for many survivors. As another individual shared, “Five years out, I still hadn’t really processed it… but I had physical reactions I couldn’t explain, and it felt like I was always exhausted.” These accounts emphasize the need for research that comprehensively measures both mental and physical health outcomes across multiple time points.

### Research priority 2: include comprehensive outcomes beyond traditional mental health metrics

Research Priority 2 emphasizes the need to expand outcome measures to include both the mental health impact of school shootings and the downstream effects, such as adverse academic and financial outcomes. This expanded approach would better capture the full range of challenges survivors face during recovery, offering a clearer picture of how these events impact their overall well-being.

#### Changes in relationship, social dynamics, and social isolation

Participants consistently reported significant disruptions in their social relationships following school shootings, identifying these changes as a critical factor affecting their mental health. A recurring theme was the dissolution of friendships leading to social isolation, particularly with individuals who had not shared the traumatic experience. This was frequently attributed to a lack of mutual understanding, with some participants expressing how difficult it became to relate to others. As one survivor noted, “People were generally going out less… there was definitely a shift… you know, especially after you’ve been affected by a shooting, it’s like a huge point that you can no longer relate to the general public with.” Another participant shared the complexity of how these social shifts compounded their trauma: “It gets really, really complicated… the friends I had… don’t like the side I’ve taken… and we’re pitted against each other by attorneys… everybody’s reacting out of trauma.” This emotional toll of disconnection was further emphasized by a mother of a survivor, who stated, “Friends that she had… can’t understand… all of those friends have all pretty much dissipated.” Such narratives underscore how trauma-induced social isolation can lead to a decline in mental health, prompting participants to call for research on the broader impact of school shootings on survivors’ social relationships and subsequent well-being.

#### Academic performance

Participants expressed varying perspectives on whether academic performance, such as grades, should be included in studies assessing the impact of school shootings. One participant argued that “the grades thing is that… they shouldn’t be taken into consideration… because, you know, the school was very lenient with grades after the shooting happened.” In contrast, another participant viewed academic metrics as valuable indicators of mental health struggles, stating, “grades do drop when they come back. That’s a mental—like they’re not focusing anymore. They’re not focusing… and they’re just in the classroom, just there, but they’re not really there, so they’re zoning out.” These differing views highlight the downstream effects of trauma on academic engagement, which can be an important proxy for mental health challenges. Survivors’ experiences suggest that while academic performance may decline, grades alone may not adequately reflect the complexities of their mental health struggles. This underscores the need to consider alternative metrics that can capture the nuances of their experiences.

#### Effects on life goals and motivation

Participants also described significant changes in life goals, with many survivors abandoning previous aspirations due to increased fears or diminished motivation. For some, the trauma of school shootings shifted their ambitions in ways that reflected their altered worldview. One mother shared about her daughter, “So, I mean, at one point in time, she wanted to be a lawyer, she wanted to be a politician… now she’s like, ‘A lawyer and a politician, mom… people would get angry at me, and they’d want to shoot [me],’” indicating how the fear of future violence redirected life choices. Another participant highlighted how motivation decreased across various areas of life: “I guess kind of on the vein of lack of motivation, like looking at attendance rates could be a more quantitative metric for that,” suggesting that academic disengagement could be a measurable outcome of trauma. This shift in life trajectories was echoed by others, as one participant reflected, “There are so many people I know that from before the shooting and after the shooting, the life trajectories they had planned completely changed.” These testimonies point to the long-term impact of school shootings on survivors’ personal and professional aspirations, with many abandoning their former goals in response to the trauma they endured.

#### Impacts on substance use and reckless behavior

Participants frequently discussed the mental health challenges they faced following the trauma in the form of substance use and reckless behavior as coping mechanisms. Many described how these behaviors became more prevalent as they struggled with the emotional aftermath of the event. One participant explained, “I think, you know, for when it first happened, all of us responded in different ways. You know, some turned to substances,” pointing to the immediate mental health effects that led some to these coping strategies. This was further reinforced by another participant, who noted, “I know that a few people kind of took a turn for the worse and took on unhealthy habits and stuff, and that was their way of self-coping the best that they could. But… that’s not something that ever made it on a research paper.” These accounts underscore how trauma can exacerbate mental health struggles, leading to behaviors such as substance use that reflect deeper emotional distress and suggest that while these coping mechanisms may offer short-term relief, they can further complicate the mental health recovery process.

#### Financial implications

Participants frequently brought attention to the financial challenges stemming from the long-term economic strain of accessing ongoing mental health support. One survivor reflected, “It got harder as I became an adult… I have spent a significant amount of money trying to access mental health help.” Another survivor shared how this need has affected their future planning, stating, “If I wasn’t so focused for the last 25 years on like, I need mental health care, it is such an important part of my life that I am going to dedicate resources that I really should be saving for retirement for, but instead, I am using that so that I can, like, get up in the mornings.” These reflections reveal the financial toll of trauma-related mental health care, which extends far beyond immediate costs.

### Research priority 3: diversify research approaches and study designs

#### Study populations

Participants stressed the importance of broadening the scope of research to include various individuals and communities impacted by school shootings. The consequences of these tragedies extend well beyond the immediate victims, affecting a wide array of people, including first responders like paramedics. One participant described groups that she thought should be included: “Paramedics, you know, the first responders and all of that, I think the effects go so far and wide. Like, people who lived right behind the school, they weren’t even on the school grounds, just neighbors, and their house got taken over by the police and set up as a hub. They witnessed the whole thing. I mean, those people needed help as well. I could list like 100 different groups.” Others spoke to how the ripple effects of firearm violence impact numerous lives, emphasizing the need for research that includes additional populations not yet commonly represented in existing studies.

The emotional needs of siblings of victims also emerged as a significant concern. Participants highlighted how often these individuals are overlooked in recovery processes. One participant shared their experience and emphasized how their brother was neglected following a severe injury, stating, “I mean, my brother was completely overlooked, because all the focus had to be on me and my recovery.” Participants discussed that the rise of school shootings has created a culture of fear among students, where threats, whether credible or not, lead to canceled classes and heightened anxiety. For siblings who witness their loved ones endure trauma, the effects manifested in their relationships and overall sense of safety as they grapple with the loss and grief. One sibling noted that, “the impact on me is different because I wasn’t physically present. Over the last couple of years, grief and loss have really played into my relationships—both with friends, romantic partners—and my general sense of safety growing up.”

One parent of a survivor shared that they struggle with their own feelings of isolation and misunderstanding. One mother expressed her difficulties in discussing her child’s experience, noting that friends who have not lived through such trauma may judge her for still grieving the trauma after two and a half years noting that “they’re like, secretly judging me, that I should be getting over this thing or whatever.” Teachers, too, are impacted, with one participant noting that “a lot of teachers have actually resigned or retired earlier than they wanted to, just because of the amounts of shooting that they’ve had witnessed in their classrooms or on their campus.”

The experiences of boys and young men in the context of school shootings emerged as another vital area for exploration. Participants in multiple interviews indicated that societal expectations surrounding masculinity often hinder emotional expression in this demographic. One account described a senior boy who faced severe panic attacks years after a shooting, illustrating how he mistook his symptoms for a heart attack. The participant shared, “he needed to get into therapy desperately for the PTSD that he has. And didn’t have the slightest clue he had.” This reflects a broader theme of stigma surrounding emotional vulnerability among boys, suggesting that they represent a particularly vulnerable population that warrants dedicated research attention.

### Study designs

#### Need for additional research methods

The participants highlighted the critical need for research that captures the complex emotional responses of individuals affected by school shootings. One survivor shared insight from gatherings with peers, noting that each person displayed unique triggers and coping mechanisms. They explained, “I love numbers, but when my daughter had 20–25 kids over, all of them from the mass [school] shooting, none responded in the same way. Each one had different triggers and responses. I don’t think you could ever put a number on that.” While quantitative data offers valuable insights, it has the potential to overlook the individual stories and granular experiences that shape recovery, necessitating both research forms. As one participant stated, “I understand the importance of science and data, but you need both. You can’t just focus on the numbers. You have to remember these are real, living people you’re studying.” Concerns were raised about research that overlooks their perspectives, with one participant stating, “The people measuring it don’t have any vested interest. They’re just looking at numbers, far removed from what actually happened.” One participant urged researchers to prioritize study approaches that incorporate the voices of those directly impacted, noting that “there needs to be more input from the communities affected by gun laws, more qualitative research, not just numbers from research.”

#### Type of school shootings

Participants expressed the need for research to focus on all types of school shootings, including non-mass incidents, which can profoundly impact survivors. One participant conveyed the intense fear and uncertainty that accompanies such events, stating, “In that moment, obviously, like, your brain goes to, like, someone got shot in the school. It’s a school shooting, like they’re going to keep going.” This highlights the psychological toll of these situations, where students face immediate threats and an unpredictable outcome. Survivors articulated feelings of invalidation when discussing non-mass shootings, noting that the perception of these incidents as less severe often leads to dismissal of their experiences. One student pointed out, “Even people within the community were kind of invalidating… invalidating people’s feelings just because of the severity of it.” Participants expressed frustration over the disproportionate attention given to mass shootings, arguing that non-mass shootings can be equally traumatic. As one survivor emphasized, “I think it’s important to focus on non-mass shootings as well, because we’re all affected by the same, you know, horrible events.” This sentiment underscores the necessity of validating the experiences of all survivors to ensure that their emotional and psychological effects are adequately acknowledged.

The lack of attention to non-mass shootings can complicate the healing process, as survivors noted feeling dismissed or unheard. One survivor remarked on the gap in information and awareness, stating, “I knew that the shooting wasn’t like other school shootings, and I didn’t really have the vocabulary to place it. But when I thought about it, I realized that shootings like this aren’t really talked about in the public sphere.” Findings showed substantial impacts on mental health for survivors regardless of the scale of the school shooting, highlighting the need for research to encompass the experiences of all survivors.

## Discussion

This study engaged school shooting survivors to identify key research areas regarding their mental health, aiming to enhance the relevance and impact of future research for this community. Centering survivor perspectives enhances our understanding of ongoing challenges facing survivors of school shootings, which should be prioritized in research that aims to inform designing and evaluating interventions and policies [14, 15]. Our findings from qualitative interviews with school shooting survivors revealed three research priorities: understanding the long-term mental health impacts across the life course, expanding outcomes beyond traditional metrics, and diversifying research approaches and populations. These priorities not only underscore the diverse experiences of survivors but also guide researchers in identifying essential areas of inquiry that can inform the development of programs aligned with community needs.

Participants emphasized the need for longitudinal research that extends beyond the acute recovery phase of school shootings. The present findings suggest that psychological challenges often persist long after the initial trauma, with recovery following a complex, non-linear trajectory marked by both progress and setbacks. As such, short-term studies may fail to capture the evolving nature of survivors’ mental health. While some research suggests that survivors of school shootings may eventually return to pre-trauma mental health levels [24], substantial evidence indicates that, for many, childhood trauma can lead to long-term mental health issues, potentially persisting into adulthood [25, 26]. Studies in other trauma-affected populations, such as veterans with PTSD, also report that recovery is not a binary process (recovered vs. not recovered) but is dynamic and non-linear [27]. This mirrors the experiences of school shooting survivors in the present study, who stressed that research must account for the fluid nature of trauma responses, including the potential for delayed psychological and physical symptoms. Despite this, the limited availability of longitudinal studies in this population—those that track participants over an extended period—hampers our understanding of long-term effects and support or intervention options among school shooting survivors. A review of mass shooting studies (non-specific to schools) found that slightly more than half of the 49 studies measured outcomes at multiple time points, and only five extended beyond two years post-shooting [28]. Similarly, a review of interpersonal firearm violence and mental health revealed that nearly half of the identified studies were cross-sectional, further highlighting the need for more research focused on long-term outcomes [29]. This research is necessary as these insights are crucial for developing interventions and support systems that adapt to survivors’ changing needs over time. Social media analyses could provide valuable, real-time data for tracking survivors’ ongoing mental health, offering insights into their experiences and well-being [30]. Additionally, exploring data from crisis tip lines may yield further understanding of survivors’ mental health over time, serving as another important source of information about their needs [31]. 

The results of our interviews also stress the importance of using diverse study designs that include survivor voices. A recent scoping review found that of all studies that examined school shootings and mental health, only 11 of the 84 included studies included qualitative findings, noting a unique gap in the literature. By capturing unique emotional experiences and coping mechanisms, which can vary among individuals, researchers can gain a more comprehensive and nuanced understanding of the impact of violence. Integrating personal narratives, via designs such as qualitative research and participatory action research, with quantitative data will further enhance insights into survivors’ experiences [14, 15]. 

There is a demonstrated need from the results of the present study to expand research on school shootings to include a broader range of outcomes beyond traditional mental health metrics. For example, disruptions in social relationships heightened feelings of isolation and interfered with survivors’ ability to rebuild support networks. Given the strong evidence linking social isolation to worsened mental health outcomes [32, 33], this issue is particularly significant, as isolation may both result from and contribute to mental health challenges following school shootings. Peer-led interventions like Psychological First Aid (PFA), which provide immediate, culturally-informed support to mitigate distress and promote adaptive coping, could be valuable in addressing this gap by fostering connection and guiding survivors toward additional resources [34]. Increased substance use among survivors reported by participants highlights the need for interventions that address both emotional and practical aspects of recovery. While trauma and substance use are well-documented in other populations including parents of children who have experience a traumatic event [35], veterans [36], and first responders [37], this relationship has not been fully explored among school shooting survivors.

The multifaceted consequences of trauma extend beyond immediate emotional responses, including a lack of motivation and financial well-being. Survivors reported a lack of both academic and general motivation following the event, a consequence that remains underexplored in this population. Research has indicated that prolonged childhood trauma is linked to decreased self-efficacy and self-esteem, which are known factors contributing to a lack of motivation [38]. This suggests that understanding the interplay between these elements is an important area for future study. Additionally, survivors highlighted the long-term financial burdens of accessing mental health care, emphasizing the need to incorporate economic factors into assessments of well-being. The allocation of limited resources to support mental health and daily functioning among our study participants revealed the profound, lasting impact of trauma on survivors’ ability to manage both their mental and financial well-being, especially as the already scarce resources often diminish after the immediate crisis phase. In a cohort of adult trauma patients, financial hardship related to medical expenses was a significant burden, with younger age identified as a predictor [39]. Given that survivors in the present study spoke of similar challenges, expanding research to explore these multidimensional impacts—particularly the interplay between financial strain from costs related to their mental health and diminished motivation—can better inform policies and support systems that address their complex, evolving needs. By considering these outcomes, researchers can develop more comprehensive interventions that integrate mental health treatment with practical resources to alleviate the broader consequences of trauma.

To effectively capture the range of individuals affected by school shootings, there is a need to diversify study populations. Survivors emphasized the importance of including participants such as siblings, first responders, parents, and teachers, who often experience trauma but are frequently overlooked in research. Current studies tend to focus primarily on direct victims, leaving gaps in understanding the emotional and psychological impacts on others. Studies have linked childhood illness to parental psychological health [40]. Research following the 2011 Norway shootings found that parents experienced anxiety and depression at three times the rate of the general population, with PTSD rates five times higher [41]. However, this topic is less studied regarding childhood trauma from US school shootings, where factors like the culture around firearms, higher endemic firearm violence rates, and different policy landscapes may lead to different psychological impacts on families in comparison to other country contexts. Understanding the impact of school shootings on parents is crucial not only for providing support to them but also because their mental health can significantly affect their children’s perceptions and reactions to such events [42]. Additionally, participants in our study highlighted the need to include other family members, such as siblings, who often face emotional challenges that receive little attention in the literature. This broader focus acknowledges that the psychological impacts of school shootings extend beyond direct victims, affecting entire families and communities.

## Limitations

This study is subject to several limitations and should be interpreted in that context. First is the potential underrepresentation of certain perspectives in our sample, particularly regarding self-identified gender, especially men, and race, which do not align with the broader demographic distribution of the general US population. This is especially important given the under-representation of Black men, who are disproportionately affected by gun violence. Their absence in this sample may have resulted in the omission of key themes, such as the connection between school shootings and outcomes like gun carrying and justice system involvement, as highlighted in other research [43]. Additionally, the study does not include other important perspectives—school personnel such as administrators and first responders—whose experiences and insights are crucial in understanding the broader impact of school shootings. However, a strength of this study is that we included participants from both mass and non-mass school shootings, as the experiences of non-mass shooting survivors are frequently overlooked in the literature. Additionally, there is potential sampling bias since most participants were affiliated with social support groups. While this approach facilitated ethical and confidential recruitment, it may have influenced the perspectives we gathered during interviews. Our research team identified this limitation in advance and opted against individual participant recruitment to prioritize respect for their experiences and anonymity, thereby minimizing the risk of inflicting further harm. Furthermore, we were unable to include survivors of elementary school shootings, which may hinder our ability to capture the full scope of the developmental impacts of school shootings on an individual’s lifecourse, particularly in terms of how early exposure to such trauma might influence later stages of cognitive, emotional, and social development.

## Conclusions

This qualitative study has outlined research priorities identified by survivors of school shootings, including investigating persistent long-term mental health challenges, assessing the impact of school shootings on social dynamics, and analyzing the financial implications of trauma for survivors and their families. To address these identified needs, researchers should prioritize asking questions that utilize diverse study designs—particularly those incorporating qualitative methods and engaging broader participant populations, such as family members and first responders—to capture the full spectrum of trauma-related impacts. Funding organizations must prioritize resources for these initiatives to support research that aligns with the needs of survivors. Ultimately, integrating these insights into research will enable the development of targeted, evidence-informed interventions that address the unique needs of all individuals affected.

## Appendix 1

### Semi-Structured interview guide

During our time together, we will be reviewing the results of a recent project we did where we looked at existing research about how school shootings affect mental health. We found several gaps in what we know about this relationship, and we are hoping to get input from all of you about these findings and how they align with how you were impacted by a school shooting so that we as researchers can do our jobs better and support the needs of the community.

As mentioned, we’ve looked into previous research about the relationship between school shootings and mental health and we’re curious what your thoughts are on these things:

1. First, we found that most studies focus on the short-term effects of shootings and often stop looking at effects within one year of the shooting. How do you think these events continue to affect people over time?
  - *Probe*: For example, in comparison to, let’s say, the first month after the shooting, in what ways does the shooting impact you now?
2. We want to find ways to help people cope better after these events, but we don’t know enough about when and how the mental health impacts occur. When thinking about yourself or your community, what personal, community, or societal factors have you experienced or observed that helped navigate the aftermath of the school shooting, either immediately after or now?
  - *Probe*: After school shootings, researchers often study specific mental health impacts, including stress, anxiety, depression, grief, substance use, and fear. What other outcomes should researchers examine among survivors of school shootings?
3. Some research found that people affected by school shootings may struggle to access mental health services, whether there not being enough providers or not enough who have the necessary training to support school shooting survivors. Can you share if this is a challenge you have noticed, and if so, what could improve access to support services?
4. Typically, researchers focus on numbers, like how many people had anxiety after the school shooting. What do you think, if anything, can be gained from hearing stories directly from survivors in their own words?
  - *Probe*: Who do you wish you could have shared your story with?
  - *Probe*: How do you hope that your stories help inform efforts to prevent future gun violence?
5. Does anyone have any additional thoughts or experiences that haven’t yet been discussed about how the school shooting you and your community experienced impacted your well-being and mental health?

## Data Availability

The datasets generated and/or analyzed during the current study are not publicly available due to the inability to fully deidentify the data. They can be made available upon reasonable request, following the IRB approval process.
